# Comparative study for haplotype block partitioning methods – Evidence from chromosome 6 of the North American Rheumatoid Arthritis Consortium (NARAC) dataset

**DOI:** 10.1371/journal.pone.0209603

**Published:** 2018-12-31

**Authors:** Mohamed N. Saad, Mai S. Mabrouk, Ayman M. Eldeib, Olfat G. Shaker

**Affiliations:** 1 Biomedical Engineering Department, Faculty of Engineering, Minia University, Minia, Egypt; 2 Biomedical Engineering Department, Faculty of Engineering, Misr University for Science and Technology (MUST), 6th of October City, Egypt; 3 Systems and Biomedical Engineering Department, Faculty of Engineering, Cairo University, Giza, Egypt; 4 Medical Biochemistry and Molecular Biology Department, Faculty of Medicine, Cairo University, Cairo, Egypt; Universidade Nova de Lisboa Instituto de Higiene e Medicina Tropical, PORTUGAL

## Abstract

Haplotype-based methods compete with “one-SNP-at-a-time” approaches on being preferred for association studies. Chromosome 6 contains most of the known genetic biomarkers for rheumatoid arthritis (RA) disease. Therefore, chromosome 6 serves as a benchmark for the haplotype methods testing. The aim of this study is to test the North American Rheumatoid Arthritis Consortium (NARAC) dataset to find out if haplotype block methods or single-locus approaches alone can sufficiently provide the significant single nucleotide polymorphisms (SNPs) associated with RA. In addition, could we be satisfied with only one method of the haplotype block methods for partitioning chromosome 6 of the NARAC dataset? In the NARAC dataset, chromosome 6 comprises 35,574 SNPs for 2,062 individuals (868 cases, 1,194 controls). Individual SNP approach and three haplotype block methods were applied to the NARAC dataset to identify the RA biomarkers. We employed three haplotype partitioning methods which are confidence interval test (CIT), four gamete test (FGT), and solid spine of linkage disequilibrium (SSLD). *P*-values after stringent Bonferroni correction for multiple testing were measured to assess the strength of association between the genetic variants and RA susceptibility. Moreover, the block size (in base pairs (bp) and number of SNPs included), number of blocks, percentage of uncovered SNPs by the block method, percentage of significant blocks from the total number of blocks, number of significant haplotypes and SNPs were used to compare among the three haplotype block methods. Individual SNP, CIT, FGT, and SSLD methods detected 432, 1,086, 1,099, and 1,322 associated SNPs, respectively. Each method identified significant SNPs that were not detected by any other method (Individual SNP: 12, FGT: 37, CIT: 55, and SSLD: 189 SNPs). 916 SNPs were discovered by all the three haplotype block methods. 367 SNPs were discovered by the haplotype block methods and the individual SNP approach. The *P*-values of these 367 SNPs were lower than those of the SNPs uniquely detected by only one method. The 367 SNPs detected by all the methods represent promising candidates for RA susceptibility. They should be further investigated for the European population. A hybrid technique including the four methods should be applied to detect the significant SNPs associated with RA for chromosome 6 of the NARAC dataset. Moreover, SSLD method may be preferred for its favored benefits in case of selecting only one method.

## Introduction

Many researchers associate RA disease with genetic biomarkers through individual SNP studies. Recently, the availability of high genomic density of SNPs allows the application of the haplotype block methods. These methods discover a group of SNPs within an associated block in only one test [1, 2].

The main advantages of haplotype block methods over individual SNP approaches are: (a) the reduction of the association testing dimension by using a single test for a block containing more than one SNP; (b) leading to power preservation and ensuring accepted false-positive rates [3]; (c) acquiring the synergy among SNPs. The main disadvantages of haplotype block methods are: (a) the more haplotypes within a block leads to a higher degree of freedom of the block ending in scaled down power; (b) each partitioning method ends up with haplotype blocks that differ from the others. Therefore, a comparison study should be performed to evaluate all partitioning methods’ performance [1, 4].

Singular SNP approaches achieve inspiring results if there should be an occurrence of monogenetic disorders (for example, sickle cell anemia). Then again, they don't achieve a similar accomplishment in complex diseases. The power of association was studied for individual SNP approaches and haplotype block methods resulting in contradictory findings. This inconsistency may be arisen from the dependency of the method’s performance on the nature of the experimented dataset itself [1, 5].

RA is a chronic autoimmune disease that is prevalent in women more than in men (with a ratio of about 3:1) [6–9]. The MHC (major histocompatibility complex) region extends on the short arm of chromosome 6 (6p21.3) from 26 to 34 Mb (mega base pair) [10]. The MHC region contains the HLA (human leukocyte antigen) region [11] which includes about 50% of the detected biomarkers for RA susceptibility. The association between HLA region and RA disease has been verified in multiethnic populations [12].

There are two objectives of this study. The first objective is comparing the association results of haplotype block methods and individual SNP approach on chromosome 6 of the NARAC dataset. The second objective is selecting the best haplotype block method suitable for the NARAC dataset (if applicable).

## Materials and methods

### Study population

The NARAC dataset consisted of 2,062 participants (1,493 females and 569 males), 868 RA patients and 1,194 healthy controls. All cases and controls were of European descent. All participants were genotyped on the HumanHap500 v1, Human Hap500 v3, HumanHap300, and HumanHap240 Illumina arrays [13]. The studied genetic variants were 35,574 SNPs included in chromosome 6. After removing 1,452 SNPs, 34,122 SNPs were retained for further analysis. The reasons for excluding the 1,452 SNPs were the biomarker checks: (a) less than 75% genotype percent, (b) less than 0.001 Hardy-Weinberg equilibrium (HWE) *P*-value or (c) less than 0.001 minor allele frequency (MAF) in the total sample.

### Materials

The chromosome 6 data file was extracted from the NARAC data file using the programming language (Perl). The chromosome 6 data file was reformatted to be ready for processing by the program (PLINK) by the statistical package (R 3.1.0). Moreover, the R language was used to extract the chromosome 6 map file from the NARAC map file (SNP ID, physical position, and chromosome number). The chromosome 6 reformatted data and map files were processed by (PLINK 1.07) and (gPLINK 2.05) programs to be ready for processing by the program (Haploview) [14].

**Fig 1:**
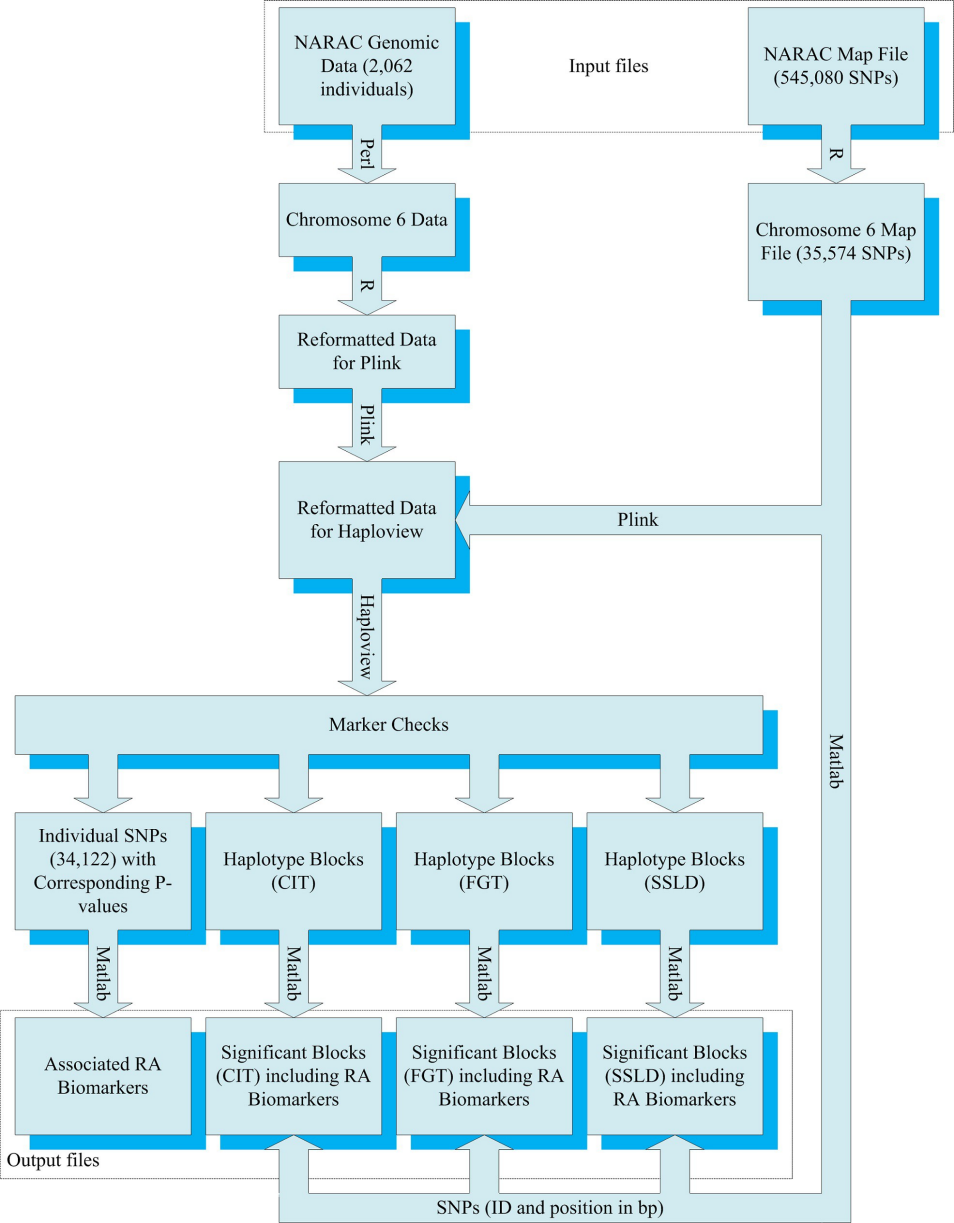
Experimental design of the proposed system.

The computer program (Haploview 4.2) was used to partition chromosome 6 into successive blocks using CIT, FGT, and SSLD methods and to calculate the corresponding *P*-value for each haplotype in each block. The default parameters for the three methods were used. In addition, the program (Haploview 4.2) was used to apply the individual SNP approach and to provide the corresponding *P*-value for each SNP [15]. The distribution of *P*-values on chromosome 6 for the individual SNP approach was presented using the Integrative Genomics Viewer (IGV 2.3.72) [16, 17]. The significant blocks and the associated SNPs were selected using the programming language (Matlab Release 2010a). Fig 1 showed a block diagram for the whole chromosomic association analysis process.

### Haplotype block methods

The haplotype blocks have been defined through more than one method. Several algorithms for haplotype partitioning have been proposed, among which the CIT, FGT, and SSLD have been implemented in HaploView 4.2. Each method differs greatly from the others in its scope of the definition of the haplotype blocks. Therefore, an objective has been arisen for comparing the results of these methods which was studied through this research paper [18].

#### Confidence interval test

A haplotype block is defined by the CIT method as a region over which a very small fraction (≤ 5%) of the measures among the informative SNP pairs are in weak linkage disequilibrium (LD). The informative SNP pairs are pairs showing strong LD or weak LD. Biological and artefactual forces in addition to recombination events are the reasons for allowing 5% of weak LD in the haplotype block. These forces could be recurrent mutation, gene conversion, or errors of the genome assembly or genotyping.

Dˊ (normalized deviation) is used to measure the LD between a pair of SNPs. CI is used to assess the reliability of the estimate of Dˊ. Strong LD is defined as the upper limit of the 90% CI is (0.98), and the lower limit is (0.7). In contrast, weak LD is defined as the upper limit of the 90% CI is (0.9), as shown in S1 Fig. The thresholds were obtained by Gabriel et al. Short blocks (2–5 SNPs) were treated with different thresholds for different populations to select the used thresholds [19].

#### Four gamete test

The FGT is a haplotype block partitioning method that assumes recombination events are not allowed within each block. When the four gametes are identified, a recombination event has been occurred. The rare gamete must be observed at a frequency greater than 0.01 to count a recombination event. The recombination events are only accepted between blocks. The FGT method differs from other haplotype block definitions that it does not require a threshold for Dˊ.

The recombination events interrupt the continuity of the testing process. When a recombination event is observed between the (k^th^) locus and any preceding locus, the locus (k-1) is considered the ending point of the tested block. The block size could be measured as the distance between the start locus and the end locus. In this situation, the locus (k) is considered the starting point to search for a new block [20].

#### Solid spine of linkage disequilibrium

The SSLD method defines the haplotype block as a region at which a (spine) of strong LD (Dˊ > 0.8) moving from one allele to the next allele along the legs of the triangle in the LD chart, as shown in S2 Fig. In other words, for each block, there must be a strong LD between (the first SNP and the last SNP) and all the intermediate SNPs. However, the intermediate SNPs should not be in strong LD with each other [15].

Table 1 represents the concept of the SSLD method. Five SNPs are tested, having the same results as in S2 Fig. Here, (SNP2 and SNP3), (SNP3 and SNP4), and (SNP2 and SNP4) are not in strong LD. All other combinations of SNPs are in strong LD. Thus, there is a haplotype block extends from SNP1 to SNP5. Table 2 shows a comparison among the three haplotype block definitions.

**Table 1:** Example on the solid spine of linkage disequilibrium (SSLD) method.

SNP#	SNP1	SNP2	SNP3	SNP4	SNP5
**SNP1**	-	0.97	0.99	0.93	0.96
**SNP2**		-	0.18	0.67	0.98
**SNP3**			-	0.03	0.94
**SNP4**				-	0.95
**SNP5**					-

**Table 2:** A comparison among haplotype block definitions.

Items	FGT	CIT	SSLD
**Recombination Event within Block**	Not Allowed	≤ 5%	Allowed only between intermediate SNPs
**Strong LD**	LD is not used	Dˊ upper limit = 0.98 Dˊ lower limit = 0.7	Dˊ > 0.8
**Weak LD**	LD is not used	Dˊ upper limit = 0.9	Dˊ ≤ 0.8
**Morphology in the LD Chart**	No recombination event between all SNPs in the block	> 95% Strong LD between all SNPs in the block	Strong LD in the legs of the LD chart

### Testing for associations with disease status

Both the individual SNP associations and the haplotype associations were measured with the aid of the *P*-values. The statistically significant SNPs were detected using their corresponding *P*-values after stringent Bonferroni correction for multiple testing (< 0.05/# of tests) [21]. The Bonferroni thresholds were 8.49 × 10^−6^ for the CIT method, 7.95 × 10^−6^ for the FGT method, 9.41 × 10^−6^ for the SSLD method, and 1.47 × 10^−6^ for the individual SNP approach where the total number of tests were 5,888 for the CIT method, 6,293 for the FGT method, 5,313 for the SSLD method, and 34,122 for the individual SNP approach.

## Results

The testing algorithms were applied on Intel Core i7-4720HQ 2.6 GHz system with 16 GB of RAM. S1 Table provided the processing time for each used program. A total working time was 181 minutes.

**Fig 2:**
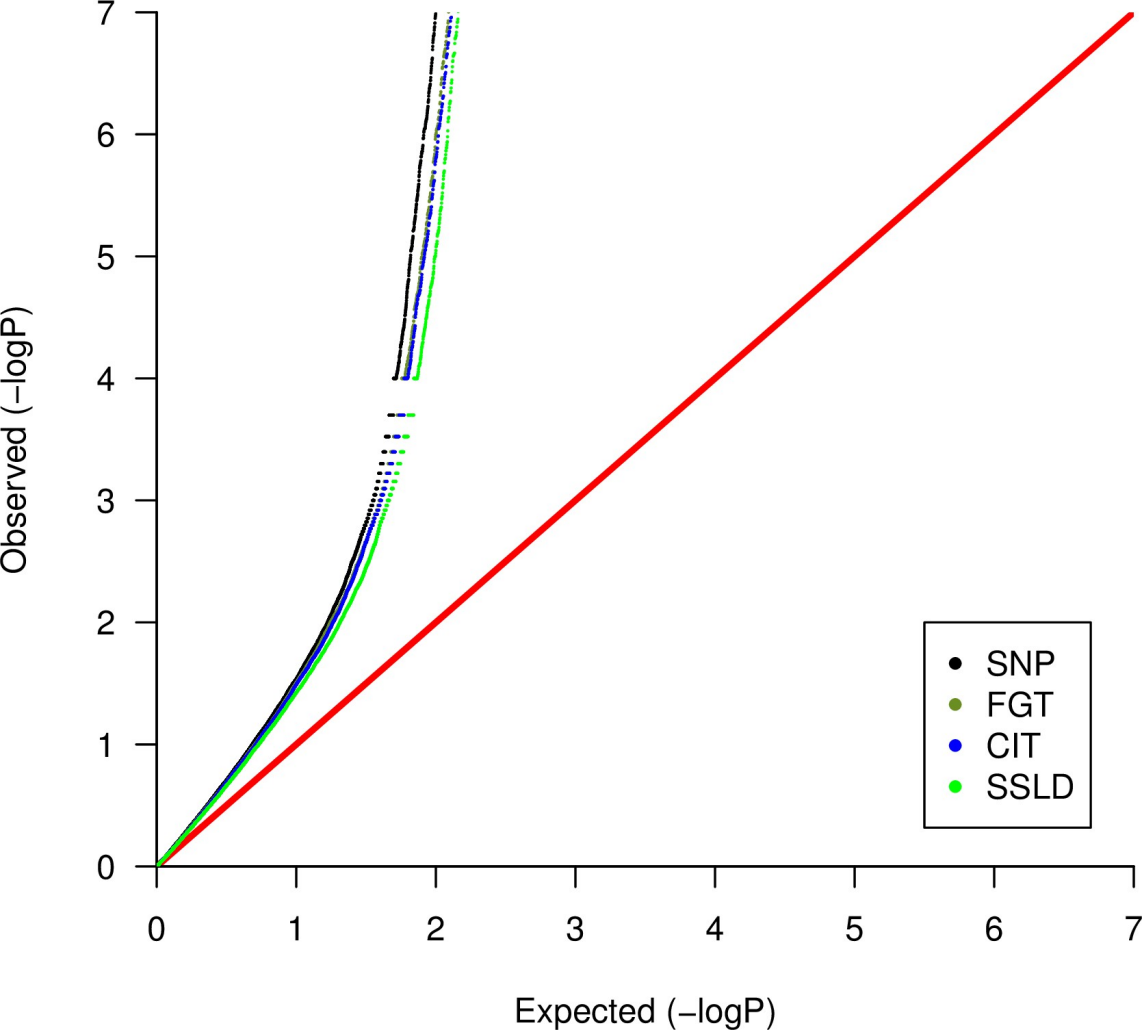
Quantile–quantile (Q–Q) plots of the chromosome 6 association results for all the used methods. Q-Q plot shows the observed distribution of–log_10_(*P*-values) on the Y-axis compared to the expected distribution of–log_10_(*P*-values) on the X-axis. The red line (X = Y) represents the null distribution. SNP refers to the individual SNP approach.

**Fig 3:**
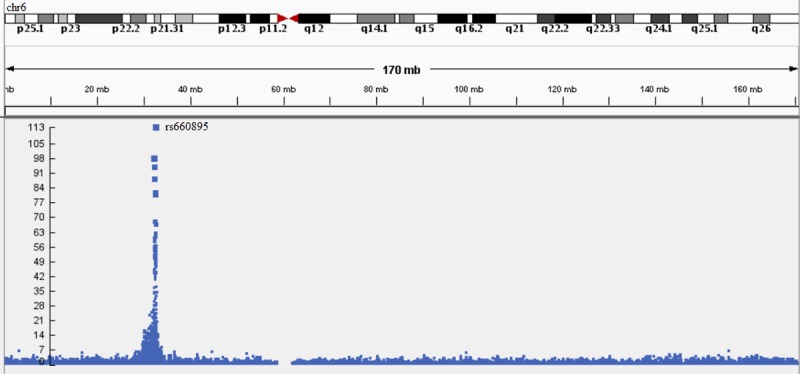
Manhattan plot for the associations between genotyped SNPs (chromosome 6 of the NARAC dataset) and RA susceptibility using individual SNP approach. The upper part represents chromosome 6 ideogram and its genomic coordinates. The Y-axis shows–log_10_(*P*-values), which represent the strength of association. The larger the point and the higher the point on the scale, the more significant the association with RA susceptibility.

The distribution of the observed *P*-values for all the used models showed evidence for population stratification (Fig 2) [22, 23]. The reason for this stratification might be the selection of chromosome 6 that contains the HLA region where many highly significant associations occur. The distribution of the *P*-values across chromosome 6 was presented in Fig 3 for the individual SNP approach. The top SNP (rs660895) in the HLA region (32,685,358 bp) had the lowest *P*-value (1.03 X 10^−113^) as previously reported in Arya et al. [24].

The results related to the haplotype block methods are shown in Table 3. The associated SNPs properties are included in S1 Spreadsheet for the CIT method, S2 Spreadsheet for the FGT method, S3 Spreadsheet for the SSLD method, and S4 Spreadsheet for the individual SNP approach.

**Table 3:** A comparison among haplotype block methods results.

Items	CIT	FGT	SSLD
**Total number of blocks**	5,888	6,293	5,313
**Maximum block size (bp)**	480,781	351,442	430,369
**Maximum significant block size (bp)**	209,236	142,115	198,772
**Median block size (bp)**	8,456	9,582	13,943
**Median significant block size (bp)**	8,671	7,447	10,122
**Minimum block size (bp)**	6	6	6
**Minimum significant block size (bp)**	25	25	76
**Median number of SNPs within each block**	3	4	5
**Median number of SNPs within each significant block**	5	5	7
**Percentage of uncovered SNPs by the block method**	19.46%	10.25%	3.3%
**Percentage of significant blocks from the total number of blocks**	2.72%	2.84%	2.73%
**Total number of significant haplotypes**	307	343	302
**Total number of significant SNPs**	1,086	1,099	1,322
**Intersection with SNPs detected by individual SNP approach (432 SNPs)**	381	387	415
**Intersection with top 50 SNPs (lowest *P*-values) detected by the individual SNP approach**	47	48	50

The SSLD significant blocks included more associated SNPs (1,322) than FGT (1,099) and CIT (1,086) blocks. Moreover, the number of the associated SNPs by the individual SNP approach was (432). The SSLD method did the best job of representing the hits of the individual SNP approach with (415) SNPs. While, the CIT method represented (381) SNPs, and the FGT method represented (387) SNPs (S5 Spreadsheet). Interestingly, the SSLD method totally represented the top 50 associated SNPs by the individual SNP approach from (*P*-values) point of view. In addition, the CIT and FGT methods represented (47) and (48) SNPs respectively from the top 50 SNPs, as shown in Table 4.

**Table 4:** The top 50 associated SNPs discovered by the individual SNP approach with the corresponding haplotype blocks.

SNP ID	Position (bp)	Assoc. Allele^a^	AAF^b^ (Case, Control)	P-value	Gene / Nearest Genes	Haplotype Block (Method, P-value, No. of SNPs in Block)	Haplotype Block Position (bp) (Start, End, Size)	Previously Studied in
rs1033500	32415360	A	0.619, 0.393	2.1 E-45	C6orf10	CIT, 3.49 E-44, 15	32415360, 32445664, 30305	[25]
						FGT, 3.12 E-44, 8	32398932, 32425613, 26682	
						SSLD, 1.48 E-97, 26	32390832, 32445664, 54832	
rs1980495	32454772	C	0.511, 0.269	3.45 E-52	C6orf10 / BTNL2	CIT, 4.08 E-59, 9	32454772, 32471794, 17023	[26, 27]
						FGT, 4.08 E-59, 9	32454772, 32471794, 17023	
						SSLD, 1.46 E-81, 3	32449376, 32456123, 6748	
rs2076530	32471794	G	0.699, 0.444	2.97 E-59	BTNL2	CIT, 4.08 E-59, 9	32454772, 32471794, 17023	[28–32]
						FGT, 4.08 E-59, 9	32454772, 32471794, 17023	
						SSLD, 4.62 E-59, 7	32462406, 32471794, 9388	
rs2395157	32456123	G	0.528, 0.276	8.58 E-60	C6orf10 / BTNL2	CIT, 4.08 E-59, 9	32454772, 32471794, 17023	[33]
						FGT, 4.08 E-59, 9	32454772, 32471794, 17023	
						SSLD, 1.46 E-81, 3	32449376, 32456123, 6748	
rs2395163	32495787	G	0.509, 0.203	1.61 E-94	BTNL2 / HLA-DRA	CIT, 5.66 E-95, 11	32495758, 32512856, 17098	[34–39]
						FGT, 4.41 E-95, 5	32491086, 32495787, 4702	
						SSLD, 4.86 E-95, 14	32491086, 32512837, 21752	
rs2395185	32541145	A	0.589, 0.315	1.01 E-68	HLA-DRA / HLA-DRB5	CIT, 6.63 E-131, 11	32541145, 32713862, 172718	[32, 36, 37, 40, 41]
						FGT, 5.2 E-72, 4	32536263, 32678378, 142116	
						SSLD, 3.47 E-77, 9	32519501, 32678378, 158878	
rs2516049	32678378	G	0.576, 0.305	2.61 E-67	HLA-DRB1 / HLA-DQA1	CIT, 6.63 E-131, 11	32541145, 32713862, 172718	[32, 33, 36, 37, 42, 43]
						FGT, 5.2 E-72, 4	32536263, 32678378, 142116	
						SSLD, 3.47 E-77, 9	32519501, 32678378, 158878	
rs2647012	32772436	G	0.837, 0.624	1.76 E-50	HLA-DQB1 / HLA-DQA2	CIT, 3.68 E-54, 31	32772436, 32786977, 14542	[32, 44, 45]
						FGT, 3.26 E-52, 28	32772436, 32778956, 6521	
						SSLD, 1.49 E-53, 20	32767856, 32777978, 10123	
rs2856717	32778286	G	0.838, 0.624	6.97 E-51	HLA-DQB1 / HLA-DQA2	CIT, 3.68 E-54, 31	32772436, 32786977, 14542	[45]
						FGT, 3.26 E-52, 28	32772436, 32778956, 6521	
						SSLD, 1.55 E-52, 17	32778015, 32787668, 9654	
rs2856725	32774716	A	0.837, 0.623	1.05 E-50	HLA-DQB1 / HLA-DQA2	CIT, 3.68 E-54, 31	32772436, 32786977, 14542	[45, 46]
						FGT, 3.26 E-52, 28	32772436, 32778956, 6521	
						SSLD, 1.49 E-53, 20	32767856, 32777978, 10123	
rs2858305	32778442	A	0.838, 0.624	6.97 E-51	HLA-DQB1 / HLA-DQA2	CIT, 3.68 E-54, 31	32772436, 32786977, 14542	[33, 45]
						FGT, 3.26 E-52, 28	32772436, 32778956, 6521	
						SSLD, 1.55 E-52, 17	32778015, 32787668, 9654	
rs3129871	32514320	C	0.835, 0.631	2.14 E-46	BTNL2 / HLA-DRA	SSLD, 5.38 E-41, 2	32512856, 32514320, 1465	[31, 46, 47]
rs3763309	32483951	A	0.507, 0.209	6.48 E-89	BTNL2 / HLA-DRA	CIT, 1.99 E-89, 8	32483951, 32491201, 7251	[35–38, 48]
						FGT, 1.46 E-87, 7	32483951, 32488240, 4290	
						SSLD, 1.46 E-87, 6	32481290, 32487361, 6072	
rs3763312	32484326	A	0.505, 0.207	1.05 E-88	BTNL2 / HLA-DRA	CIT, 1.99 E-89, 8	32483951, 32491201, 7251	[35, 36, 39, 46]
						FGT, 1.46 E-87, 7	32483951, 32488240, 4290	
						SSLD, 1.46 E-87, 6	32481290, 32487361, 6072	
rs3817963	32476065	G	0.534, 0.294	1.23 E-54	BTNL2	CIT, 1.07 E-54, 3	32474399, 32476065, 1667	[31, 37, 39, 49–52]
						FGT, 2.51 E-42, 4	32476065, 32481676, 5612	
						SSLD, 1.54 E-54, 4	32474399, 32477466, 3068	
rs3817973	32469089	A	0.698, 0.440	5.48 E-61	C6orf10 / BTNL2	CIT, 4.08 E-59, 9	32454772, 32471794, 17023	[31, 32, 39]
						FGT, 4.08 E-59, 9	32454772, 32471794, 17023	
						SSLD, 4.62 E-59, 7	32462406, 32471794, 9389	
rs3957148	32790115	G	0.277, 0.094	1.88 E-53	HLA-DQB1 / HLA-DQA2	CIT, 6.72 E-54, 7	32788906, 32790115, 1210	[53, 54]
						FGT, 2.32 E-53, 4	32789654, 32790286, 633	
						SSLD, 6.72 E-54, 7	32788906, 32790115, 1210	
rs4424066	32462406	G	0.698, 0.440	1.18 E-60	C6orf10 / BTNL2	CIT, 4.08 E-59, 9	32454772, 32471794, 17023	[32, 55]
						FGT, 4.08 E-59, 9	32454772, 32471794, 17023	
						SSLD, 4.62 E-59, 7	32462406, 32471794, 9389	
rs477515	32677669	A	0.575, 0.304	2.72 E-67	HLA-DRB1 / HLA-DQA1	CIT, 6.63 E-131, 11	32541145, 32713862, 172718	[32, 36, 42, 56]
						FGT, 5.2 E-72, 4	32536263, 32678378, 142116	
						SSLD, 3.47 E-77, 9	32519501, 32678378, 158878	
rs5000634	32771542	G	0.614, 0.382	1.09 E-48	HLA-DQB1 / HLA-DQA2	CIT, 2.62 E-81, 3	32767856, 32771829, 3974	[32, 33, 57]
						FGT, 1.03 E-48, 4	32766602, 32771829, 5228	
						SSLD, 1.49 E-53, 20	32767856, 32777978, 10123	
rs547077	32397296	G	0.622, 0.397	3.98 E-46	C6orf10	CIT, 3.14 E-98, 18	32332366, 32406350, 73985	[39]
						FGT, 1.5 E-98, 15	32332366, 32397296, 64931	
						SSLD, 1.48 E-97, 26	32390832, 32445664, 54833	
rs6457617	32771829	A	0.803, 0.508	2.55 E-82	HLA-DQB1 / HLA-DQA2	CIT, 2.62 E-81, 3	32767856, 32771829, 3974	[32, 33, 36, 43, 48, 58–75]
						FGT, 1.03 E-48, 4	32766602, 32771829, 5228	
						SSLD, 1.49 E-53, 20	32767856, 32777978, 10123	
rs660895	32685358	G	0.529, 0.192	1.03 E-113	HLA-DRB1 / HLA-DQA1	CIT, 6.63 E-131, 11	32541145, 32713862, 172718	[33, 35–43, 46, 47, 51, 60, 65, 68, 71, 76–92]
						FGT, 1.84 E-113, 8	32680229, 32713862, 33634	
						SSLD, 8.37 E-108, 11	32680229, 32760295, 80067	
rs6903608	32536263	A	0.882, 0.688	2.84 E-48	HLA-DRA / HLA-DRB5	FGT, 5.2 E-72, 4	32536263, 32678378, 142116	[32, 33, 40, 55]
						SSLD, 3.47 E-77, 9	32519501, 32678378, 158878	
rs6910071	32390832	G	0.506, 0.195	1.25 E-98	C6orf10	CIT, 3.14 E-98, 18	32332366, 32406350, 73985	[33, 35–40, 43, 46–48, 50, 65, 68, 71, 79, 86–89, 93, 94]
						FGT, 1.5 E-98, 15	32332366, 32397296, 64931	
						SSLD, 1.48 E-97, 26	32390832, 32445664, 54833	
rs6932542	32488240	G	0.769, 0.527	1.06 E-56	BTNL2 / HLA-DRA	CIT, 1.99 E-89, 8	32483951, 32491201, 7251	[33, 95]
						FGT, 1.46 E-87, 7	32483951, 32488240, 4290	
						SSLD, 4.87 E-53, 3	32487714, 32488240, 527	
rs7192	32519624	C	0.826, 0.608	1.68 E-51	HLA-DRA	CIT, 2.05 E-25, 3	32519501, 32521295, 1795	[31, 33, 39, 46, 96, 97]
						FGT, 2.05 E-25, 3	32519501, 32521295, 1795	
						SSLD, 3.47 E-77, 9	32519501, 32678378, 158878	
rs9268528	32491086	G	0.596, 0.365	8.28 E-49	BTNL2 / HLA-DRA	CIT, 1.99 E-89, 8	32483951, 32491201, 7251	[98–100]
						FGT, 4.41 E-95, 5	32491086, 32495787, 4702	
						SSLD, 4.86 E-95, 14	32491086, 32512837, 21752	
rs9268542	32492699	G	0.597, 0.371	1.56 E-46	BTNL2 / HLA-DRA	FGT, 4.41 E-95, 5	32491086, 32495787, 4702	[98, 99]
						SSLD, 4.86 E-95, 14	32491086, 32512837, 21752	
rs9268832	32535767	G	0.819, 0.597	1.82 E-52	HLA-DRA / HLA-DRB5	CIT, 8.33 E-54, 2	32522251, 32535767, 13517	[32, 39, 41, 96]
						FGT, 8.33 E-54, 2	32522251, 32535767, 13517	
						SSLD, 3.47 E-77, 9	32519501, 32678378, 158878	
rs9275224	32767856	G	0.804, 0.511	1.74 E-81	HLA-DQB1 / HLA-DQA2	CIT, 2.62 E-81, 3	32767856, 32771829, 3974	[32, 36, 39, 99, 101]
						FGT, 1.03 E-48, 4	32766602, 32771829, 5228	
						SSLD, 1.49 E-53, 20	32767856, 32777978, 10123	
rs9275312	32773706	G	0.297, 0.121	7.05 E-45	HLA-DQB1 / HLA-DQA2	CIT, 3.68 E-54, 31	32772436, 32786977, 14542	[33, 41]
						FGT, 3.26 E-52, 28	32772436, 32778956, 6521	
						SSLD, 1.49 E-53, 20	32767856, 32777978, 10123	
rs9275371	32776274	G	0.488, 0.259	2.57 E-47	HLA-DQB1 / HLA-DQA2	CIT, 3.68 E-54, 31	32772436, 32786977, 14542	[46, 99]
						FGT, 3.26 E-52, 28	32772436, 32778956, 6521	
						SSLD, 1.49 E-53, 20	32767856, 32777978, 10123	
rs9275374	32776504	A	0.482, 0.246	1.69 E-55	HLA-DQB1 / HLA-DQA2	CIT, 3.68 E-54, 31	32772436, 32786977, 14542	[45, 47, 102]
						FGT, 3.26 E-52, 28	32772436, 32778956, 6521	
						SSLD, 1.49 E-53, 20	32767856, 32777978, 10123	
rs9275383	32776824	A	0.261, 0.085	6.20 E-50	HLA-DQB1 / HLA-DQA2	CIT, 3.68 E-54, 31	32772436, 32786977, 14542	[103]
						FGT, 3.26 E-52, 28	32772436, 32778956, 6521	
						SSLD, 1.49 E-53, 20	32767856, 32777978, 10123	
rs9275388	32777062	G	0.482, 0.241	7.21 E-57	HLA-DQB1 / HLA-DQA2	CIT, 3.68 E-54, 31	32772436, 32786977, 14542	[33, 45]
						FGT, 3.26 E-52, 28	32772436, 32778956, 6521	
						SSLD, 1.49 E-53, 20	32767856, 32777978, 10123	
rs9275390	32777134	G	0.482, 0.246	1.69 E-55	HLA-DQB1 / HLA-DQA2	CIT, 3.68 E-54, 31	32772436, 32786977, 14542	[47, 51, 57, 64, 75, 99, 104]
						FGT, 3.26 E-52, 28	32772436, 32778956, 6521	
						SSLD, 1.49 E-53, 20	32767856, 32777978, 10123	
rs9275393	32777417	A	0.482, 0.246	2.72 E-55	HLA-DQB1 / HLA-DQA2	CIT, 3.68 E-54, 31	32772436, 32786977, 14542	[45, 99]
						FGT, 3.26 E-52, 28	32772436, 32778956, 6521	
						SSLD, 1.49 E-53, 20	32767856, 32777978, 10123	
rs9275406	32777933	A	0.482, 0.246	1.38 E-55	HLA-DQB1 / HLA-DQA2	CIT, 3.68 E-54, 31	32772436, 32786977, 14542	[41, 45, 51, 105]
						FGT, 3.26 E-52, 28	32772436, 32778956, 6521	
						SSLD, 1.49 E-53, 20	32767856, 32777978, 10123	
rs9275407	32778015	A	0.483, 0.245	9.06 E-56	HLA-DQB1 / HLA-DQA2	CIT, 3.68 E-54, 31	32772436, 32786977, 14542	[45, 99]
						FGT, 3.26 E-52, 28	32772436, 32778956, 6521	
						SSLD, 1.55 E-52, 17	32778015, 32787668, 9654	
rs9275408	32778088	G	0.463, 0.231	6.75 E-52	HLA-DQB1 / HLA-DQA2	CIT, 3.68 E-54, 31	32772436, 32786977, 14542	-
						FGT, 3.26 E-52, 28	32772436, 32778956, 6521	
						SSLD, 1.55 E-52, 17	32778015, 32787668, 9654	
rs9275418	32778222	G	0.482, 0.246	1.4 E-55	HLA-DQB1 / HLA-DQA2	CIT, 3.68 E-54, 31	32772436, 32786977, 14542	[45, 106–108]
						FGT, 3.26 E-52, 28	32772436, 32778956, 6521	
						SSLD, 1.55 E-52, 17	32778015, 32787668, 9654	
rs9275424	32778554	G	0.482, 0.246	1.4 E-55	HLA-DQB1 / HLA-DQA2	CIT, 3.68 E-54, 31	32772436, 32786977, 14542	[45, 99]
						FGT, 3.26 E-52, 28	32772436, 32778956, 6521	
						SSLD, 1.55 E-52, 17	32778015, 32787668, 9654	
rs9275425	32778852	A	0.477, 0.244	5.74 E-54	HLA-DQB1 / HLA-DQA2	CIT, 3.68 E-54, 31	32772436, 32786977, 14542	[45, 99]
						FGT, 3.26 E-52, 28	32772436, 32778956, 6521	
						SSLD, 1.55 E-52, 17	32778015, 32787668, 9654	
rs9275427	32778893	A	0.482, 0.246	1.86 E-55	HLA-DQB1 / HLA-DQA2	CIT, 3.68 E-54, 31	32772436, 32786977, 14542	[45]
						FGT, 3.26 E-52, 28	32772436, 32778956, 6521	
						SSLD, 1.55 E-52, 17	32778015, 32787668, 9654	
rs9275428	32778956	G	0.482, 0.247	3.3 E-55	HLA-DQB1 / HLA-DQA2	CIT, 3.68 E-54, 31	32772436, 32786977, 14542	[45, 99, 109]
						FGT, 3.26 E-52, 28	32772436, 32778956, 6521	
						SSLD, 1.55 E-52, 17	32778015, 32787668, 9654	
rs9275439	32779499	G	0.479, 0.245	9.76 E-55	HLA-DQB1 / HLA-DQA2	CIT, 3.68 E-54, 31	32772436, 32786977, 14542	[41, 45, 99]
						FGT, 2.66 E-54, 2	32779081, 32779499, 419	
						SSLD, 1.55 E-52, 17	32778015, 32787668, 9654	
rs9275555	32785066	A	0.475, 0.222	3.04 E-63	HLA-DQB1 / HLA-DQA2	CIT, 3.68 E-54, 31	32772436, 32786977, 14542	[45, 46]
						FGT, 3.19 E-61, 3	32785066, 32786977, 1912	
						SSLD, 1.55 E-52, 17	32778015, 32787668, 9654	
rs9275572	32786977	G	0.822, 0.599	7.07 E-53	HLA-DQB1 / HLA-DQA2	CIT, 3.68 E-54, 31	32772436, 32786977, 14542	[40, 45, 110]
						FGT, 3.19 E-61, 3	32785066, 32786977, 1912	
						SSLD, 1.55 E-52, 17	32778015, 32787668, 9654	
rs9275595	32789333	G	0.462, 0.218	2.59 E-61	HLA-DQB1/ HLA-DQA2	CIT, 6.72 E-54, 7	32788906, 32790115, 1210	[39, 40, 45, 104, 111]
						SSLD, 6.72 E-54, 7	32788906, 32790115, 1210	

^a^Assoc. Allele: Associated Allele.

^b^AAF: Associated Allele Frequency.

From Table 4, the intergenic region between HLA-DQB1 and HLA-DQA2 –at the p21.32 band of chromosome 6 (MHC class II)–contained blocks with the largest number of RA-associated biomarkers by the individual SNP approach. Within this region, the SSLD method had two blocks with thirteen (*P*-value = 1.49 X 10^−53^, no. of SNPs = 20) and twelve (*P*-value = 1.55 X 10^−52^, no. of SNPs = 17) biomarkers. The CIT method was represented by a block (*P*-value = 3.68 X 10^−54^, no. of SNPs = 31) having twenty-two biomarkers. The FGT block (*P*-value = 3.26 X 10^−52^, no. of SNPs = 28) was detected containing nineteen biomarkers.

## Discussion and conclusions

In this study, 34,122 SNPs were used to examine the association with RA susceptibility in the NARAC dataset. The examined SNPs belonged to chromosome 6. The surveyed SNPs on chromosome 6 of the NARAC dataset were dense enough for the application of the haplotype block methods. Four methods were applied to assign the associations (three haplotype block methods and individual SNP approach). The three used haplotype block methods were CIT, FGT, and SSLD. The individual SNP analysis was to point to chromosome regions that are genetically linked to the disease. The haplotype block methods were to further expand from the single SNPs with the strongest signal to the actual causal variants [112].

The aim of this study is to test the NARAC dataset to find out if haplotype block methods or single-locus approach alone can sufficiently provide the significant biomarkers associated with RA. Our research failed to select the best method as each method yielded significant results that were not detected using any of the other methods. S2 Table showed the SNP IDs that were uniquely identified by each method. The individual SNP, CIT, FGT, and SSLD methods exclusively detected 12, 55, 37, and 189 SNPs, respectively. Our findings were in line with Shim et al. conclusion (but they didn’t test the SSLD method) that both the individual SNP approach and the haplotype block methods should be applied side by side to discover the valuable associations in the NARAC dataset [5].

In addition, the 367 SNPs- that were significantly associated with RA susceptibility by the individual SNP approach and the haplotype block methods- represent potent candidates for further investigations (S6 Spreadsheet). The three haplotype block methods were able to detect 916 associated SNPs in common. The SSLD method detected more significant SNPs (1,322) than CIT (1,086), FGT (1,099), and individual SNP (432) methods. This observation could be understood, as the SSLD does not take into account the LD between the intermediate SNPs. Therefore, the SSLD method is the lowest conservative method in including SNPs inside it’s blocks.

The block similarity for the three applied methods were shown in Table 5. The similarity measure represented the intersected SNPs divided by the union SNPs for the two studied methods. The highest block similarity was between the CIT method and the FGT method. While, the lowest block similarity was between the CIT method and the SSLD method. The results showed that the FGT method had the most similarity with the other methods.

**Table 5:** Block similarity among the used haplotype block methods.

Block Method	CIT	FGT	SSLD
**CIT**	1	0.76	0.71
**FGT**		1	0.74
**SSLD**			1

**Fig 4:**
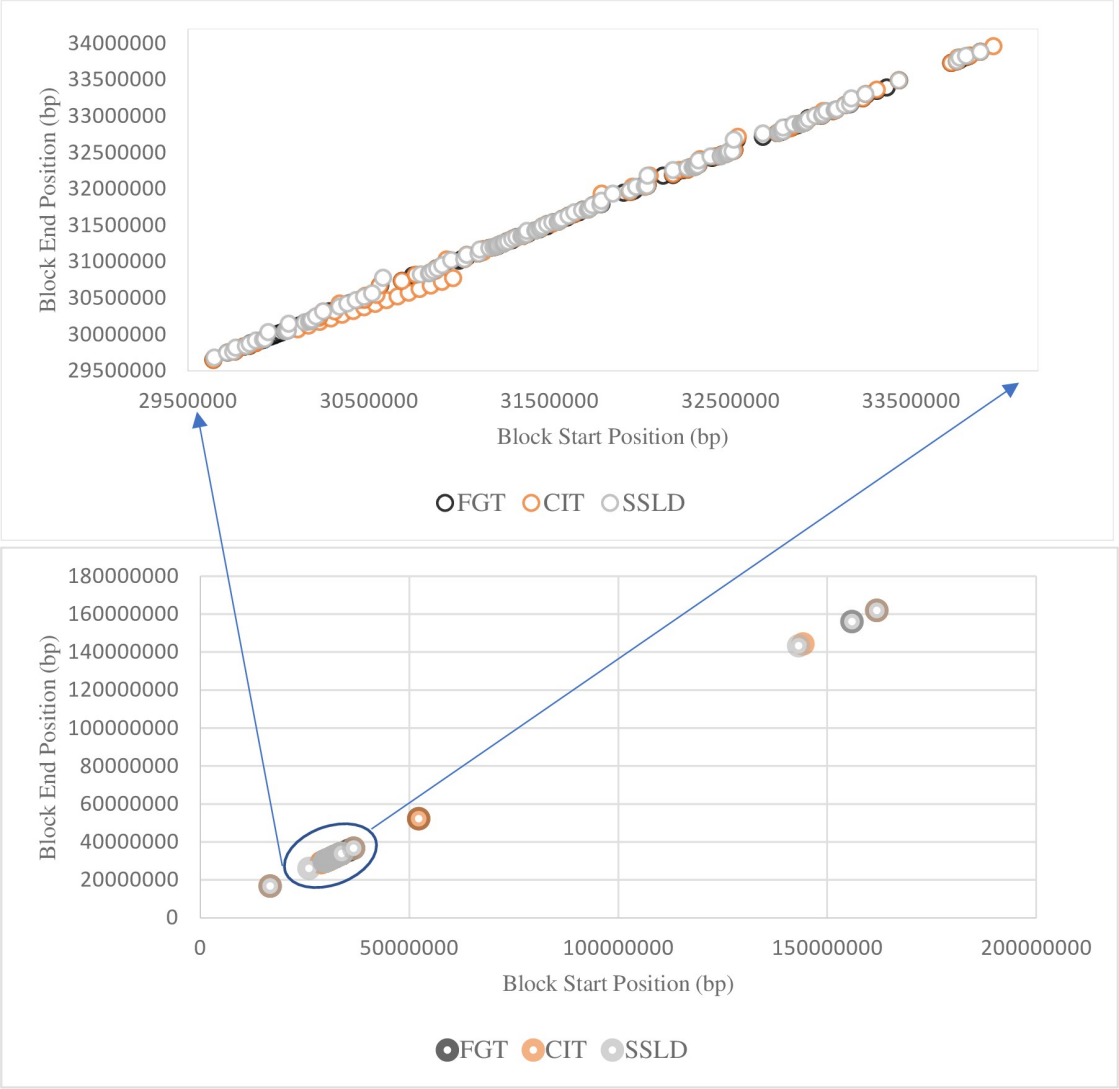
Comparison of the haplotype blocks obtained by the three methods. Each circle conistituted a significant haplotype block associated with RA susceptibility. The plot area represented chromosome 6, then zooming in on the MHC region.

Fig 4 demonstrated the overlapping of the significant blocks resulted from the three haplotype block methods. Nearly, all chromosome 6 regions that were associated with RA were in blocks that were detected by more than one method. Most of the haplotype blocks that showed significant associations with RA disease were in the MHC region or near it (+ 3 Mb). Most of the 916 SNPs that were detected by the three block methods were in the MHC region. These outcomes confirmed the strong association between the MHC region and RA susceptibility.

For further analysis of the results, a comparison between the properties of the SNPs that were detected by all the methods (intersection) and that were uniquely detected by one method (unique) was shown in Table 6. The properties of all the associated SNPs detected by the corresponding method (reference) were added to Table 6 for more clarification. For all the methods, the *P*-values of the (intersection) were lower than that of the (reference) and more lower than that of the (unique). This observation confirmed that the 367 SNPs (intersection) represents potent candidates for further investigations. For the CIT and FGT methods, the median block size (bp) of the (unique) was greater than that of the (intersection) and more greater than the (reference). However, this observation might be due to the small number of blocks representing the (unique) in comparison to the (intersection) and the (reference). While, when the number of blocks representing the (unique) (53) was sufficiently comparable to that of the (intersection) (104) and the (reference) (145) in the SSLD method, the median block size (bp) of the (intersection) was greater than that of the (unique) and more greater than that of the (reference).

**Table 6:** A comparison between the associated SNPs detected with different categories.

Method	Items	Significant SNPs detected by all methods (intersection)	All significant SNPs detected by a method (reference)	Significant SNPs detected by only one method (unique)
**CIT**	**# SNPs**	367	1,086	55
	**# blocks**	127	160	8
	**Average *P*-values**	1.63 E-07	7.20 E-07	3.33 E-06
	**Median Block Size (#SNPs)**	5	5	7
	**Median Block Size (BP)**	9,755	8,671	17,021
**FGT**	**# SNPs**	367	1,099	37
	**# blocks**	134	179	12
	**Average *P*-values**	1.37 E-07	5.6 E-07	7.84 E-07
	**Median Block Size (#SNPs)**	6	5	7
	**Median Block Size (BP)**	9,227	7,447	11,394
**SSLD**	**# SNPs**	367	1,322	189
	**# blocks**	104	145	53
	**Average *P*-values**	1.86 E-07	6.57 E-07	9.34 E-07
	**Median Block Size (#SNPs)**	9	7	8
	**Median Block Size (BP)**	17,054	10,122	11,604
**Individual SNP**	**# SNPs**	367	432	12
	**Average *P*-values**	1.25 E-07	1.35 E-07	3.26 E-07

Some associated SNPs were discovered using all the methods but others were observed by only one method. This finding might be explained by some reasons. For the associations that were observed using individual SNP approach only, it may be that only one SNP represent strong LD with the causal SNP. Therefore, studying haplotypes could decrease the power of association as they consist of several SNPs.

For the associations that were observed using the haplotype block methods only, Individual SNP approach required approximately 83% more tests than the haplotype block methods. Consequently, the Bonferroni correction was more severe for the individual SNP approach. Moreover, the haplotype block methods were able to capture the interactions among many causal SNPs. In addition, haplotypes could capture rare alleles that individual SNPs may not detect. This reason could be clarified as the power to observe associations is maximized when the frequencies of the studied biomarker and the causal SNP are similar.

For the associations that were observed using a haplotype block method but not by the other haplotype block methods, each method differs greatly from the others in its scope of the definition of the haplotype blocks. At last, we conclude that the application of the individual SNP approach and the three haplotype block methods altogether on chromosome 6 of the NARAC dataset will in turn maximize the system’s ability for discovering crucial associations. In case of selecting one method, the SSLD would be the most appropriate method for the NARAC dataset. The SSLD method has valuable advantages such as the highest genomic coverage, the largest minimum, median, and maximum significant block sizes, the biggest number of significant SNPs included in blocks, and the biggest number of associated SNPs discovered exclusively by a method.

The limitations of this study are as follows: a) the effects of population stratification were not accounted for; b) a replication study in other datasets was not performed. In addition, further investigations of other haplotype block methods, such as hidden Markov model [113, 114], dynamic programming-based algorithm [115–119], wavelet decomposition [120], greedy algorithm [121], minimum description length [122, 123], spatial correlation of SNPs [124], sequence kernel association tests [125], and block entropy [126] should be applied and compared to show the effect of the changes in block partitions on the resulting associated biomarkers.

## Supporting information

S1 Fig The confidence interval test (CIT) method showing the definition of strong LD and weak LD. (TIF)

S2 Fig LD block structure as found by the solid spine of linkage disequilibrium (SSLD) method. (TIF)

S1 Table The processing time for each performed step. (DOCX)

S2 Table SNP IDs that were uniquely identified by each method. (DOCX)

S1 Spreadsheet Properties for the associated SNPs using CIT method. Sheet1 “ID” represents SNPs IDs, and each row represents a block. Sheet2 “Bp” represents SNPs physical positions in base pairs, and each row represents a block. Sheet3 “No. of SNPs in Block” represents the number of SNPs in each block. Sheet4 “Start-Stop”–first column represents blocks start physical positions in base pairs. Sheet4 “Start-Stop”–second column represents blocks end physical positions in base pairs. Sheet4 “Start-Stop”–third column represents blocks sizes in base pairs. Sheet5 “Block no.” represents the block numbers (positions) from all blocks partitioned by CIT method. Sheet6 “*P*-values” represents the *P*-values of the blocks.
(XLS)

S2 Spreadsheet Properties for the associated SNPs using FGT method. Sheet1 “ID” represents SNPs IDs, and each row represents a block. Sheet2 “Bp” represents SNPs physical positions in base pairs, and each row represents a block. Sheet3 “No. of SNPs in Block” represents the number of SNPs in each block. Sheet4 “Start-Stop”–first column represents blocks start physical positions in base pairs. Sheet4 “Start-Stop”–second column represents blocks end physical positions in base pairs. Sheet4 “Start-Stop”–third column represents blocks sizes in base pairs. Sheet5 “Block no.” represents the block numbers (positions) from all blocks partitioned by FGT method. Sheet6 “*P*-values” represents the *P*-values of the blocks.
(XLS)

S3 Spreadsheet Properties for the associated SNPs using SSLD method. Sheet1 “ID” represents SNPs IDs, and each row represents a block. Sheet2 “Bp” represents SNPs physical positions in base pairs, and each row represents a block. Sheet3 “No. of SNPs in Block” represents the number of SNPs in each block. Sheet4 “Start-Stop”–first column represents blocks start physical positions in base pairs. Sheet4 “Start-Stop”–second column represents blocks end physical positions in base pairs. Sheet4 “Start-Stop”–third column represents blocks sizes in base pairs. Sheet5 “Block no.” represents the block numbers (positions) from all blocks partitioned by SSLD method. Sheet6 “*P*-values” represents the *P*-values of the blocks.
(XLS)

S4 Spreadsheet Properties for the associated SNPs using individual SNP approach. Sheet1 “ID” represents SNPs IDs. Sheet2 “Bp” represents SNPs physical positions in base pairs. Sheet6 “*P*-values” represents the *P*-values of the SNPs.
(XLS)

S5 Spreadsheet Intersection between haplotype methods and individual SNP approach. Sheet1 “CIT” represents SNPs IDs detected by both CIT method and Individual SNP Approach. Sheet2 “FGT” represents SNPs IDs detected by both FGT method and Individual SNP Approach. Sheet3 “SSLD” represents SNPs IDs detected by both SSLD method and Individual SNP Approach.
(XLS)

S6 Spreadsheet The SNPs IDs identified by all the used methods. (XLS)

## Abbreviations

BP: Base Pair
CIT: Confidence Interval Test
Dˊ: Normalized Deviation
FGT: Four Gamete Test
HLA: Human Leukocyte Antigen
HWE: Hardy-Weinberg Equilibrium
IGV: Integrative Genomics Viewer
LD: Linkage Disequilibrium
MAF: Minor Allele Frequency
Mb: Mega Base Pair
MHC: Major Histocompatibility Complex
NARAC: North American Rheumatoid Arthritis Consortium
Q-Q: Quantile-Quantile
RA: Rheumatoid Arthritis
SNP: Single Nucleotide Polymorphism
SSLD: Solid Spine of Linkage Disequilibrium
