# Supplementary figures and images for: Comparative study for haplotype block partitioning methods – Evidence from chromosome 6 of the North American Rheumatoid Arthritis Consortium (NARAC) dataset
Source: PLoS One. 2018 Dec 31;13(12):e0209603. doi: 10.1371/journal.pone.0209603 (PMC6312333; doi:10.1371/journal.pone.0209603)

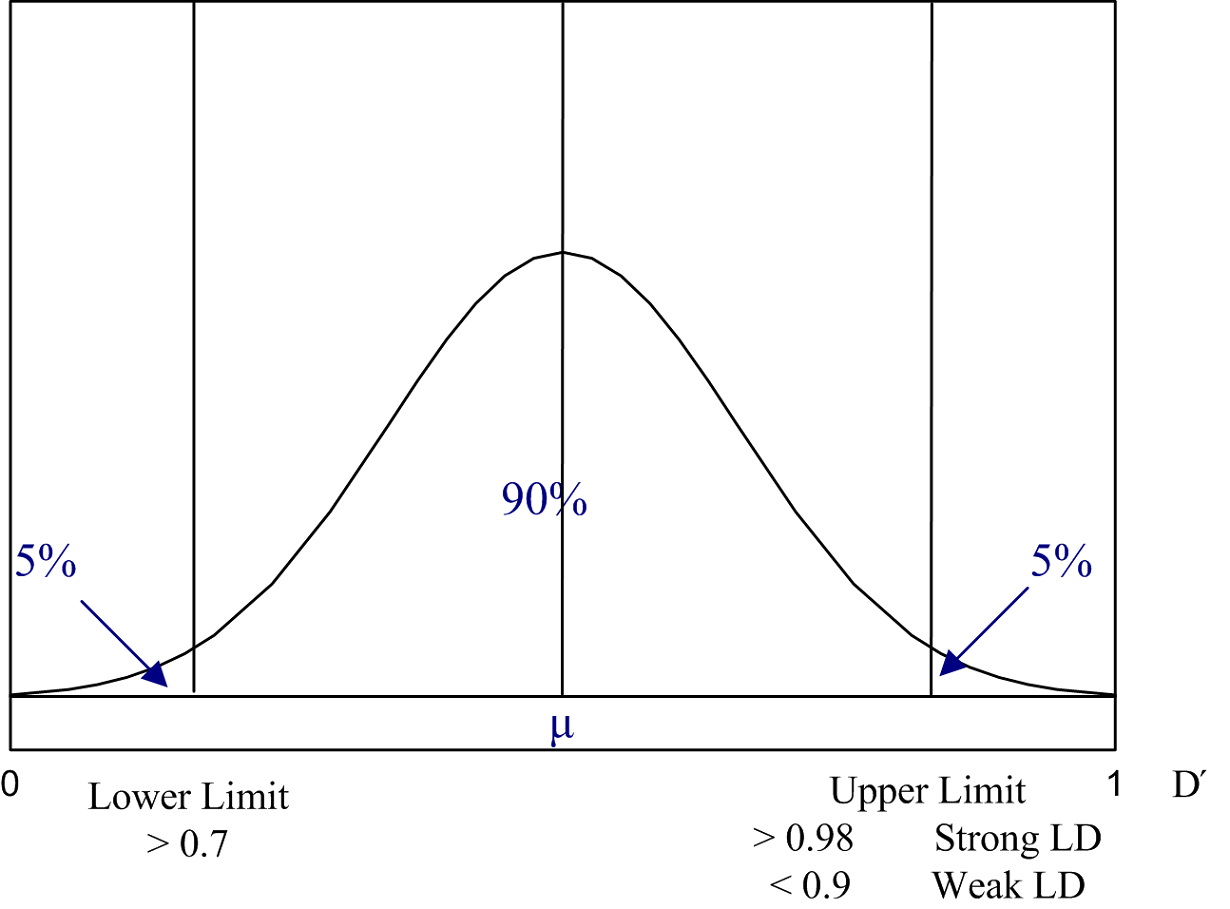

Supplement: S1 Fig — (TIF) [file pone.0209603.s001.tif]

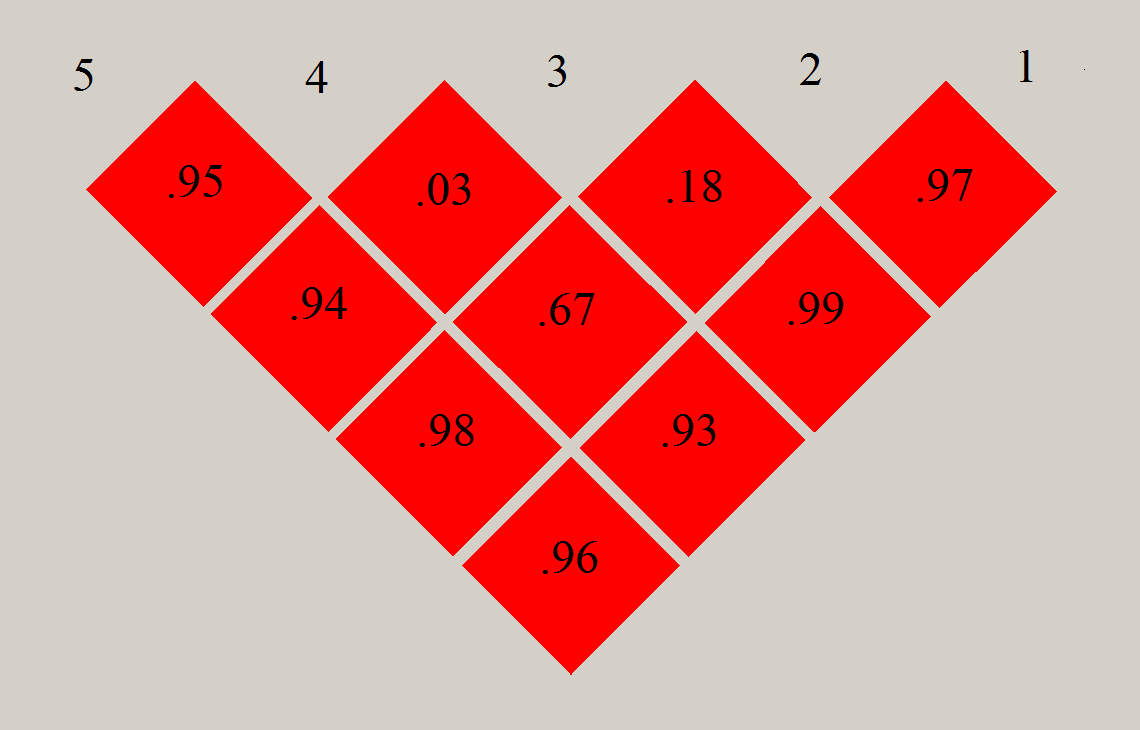

Supplement: S2 Fig — (TIF) [file pone.0209603.s002.tif]
