# Supplementary material for: Comparative study for haplotype block partitioning methods – Evidence from chromosome 6 of the North American Rheumatoid Arthritis Consortium (NARAC) dataset
Source: PLoS One. 2018 Dec 31;13(12):e0209603. doi: 10.1371/journal.pone.0209603 (PMC6312333; doi:10.1371/journal.pone.0209603)
Supplement: S1 Table — (DOCX) [file pone.0209603.s003.docx]

| **Program** | **Function** | **Processing time (min.)** |
| --- | --- | --- |
| **Perl** | Extracting chromosome 6 from the NARAC dataset | 3 |
| **R** | Reformatting data file | 5 |
| **R** | Splitting chromosome 6 marker information and reformatting map file | 5 |
| **PLINK and gPLINK** | Preparation of data and map files for Haploview program | 4 |
| **Haploview** | CIT block partitioning | 44 |
| **Haploview** | FGT block partitioning | 39 |
| **Haploview** | SSLD block partitioning | 48 |
| **Matlab** | Selecting the significant SNPs for CIT | 10 |
| **Matlab** | Selecting the significant SNPs for FGT | 11 |
| **Matlab** | Selecting the significant SNPs for SSLD | 12 |
