# Supplementary material for: Comparative study for haplotype block partitioning methods – Evidence from chromosome 6 of the North American Rheumatoid Arthritis Consortium (NARAC) dataset
Source: PLoS One. 2018 Dec 31;13(12):e0209603. doi: 10.1371/journal.pone.0209603 (PMC6312333; doi:10.1371/journal.pone.0209603)
Supplement: S2 Table — (DOCX) [file pone.0209603.s004.docx]

| **Individual SNP** | **CIT** | | **FGT** | **SSLD** | | | |
| --- | --- | --- | --- | --- | --- | --- | --- |
| rs11242834 | rs1016472 | rs9257403 | rs11156028 | rs1003979 | rs2294472 | rs3130350 | rs7746105 |
| rs12111032 | rs11155329 | rs9257425 | rs11967206 | rs1012411 | rs2294478 | rs3130573 | rs7748167 |
| rs2185955 | rs1476016 | rs9295794 | rs11967812 | rs1044429 | rs2328388 | rs3130604 | rs7749342 |
| rs2844795 | rs16869652 | rs9295967 | rs12664430 | rs10484431 | rs2394401 | rs3130647 | rs7750269 |
| rs3129882 | rs1883329 | rs9348821 | rs1894406 | rs10484432 | rs2395349 | rs3130653 | rs7752075 |
| rs3130048 | rs2071788 | rs9461924 | rs2016520 | rs1058026 | rs2395352 | rs3130696 | rs7752766 |
| rs3830041 | rs2071789 |  | rs2038068 | rs1061535 | rs2395365 | rs3130922 | rs7753935 |
| rs3873380 | rs2071790 |  | rs2071535 | rs10946999 | rs2516415 | rs3130931 | rs7754200 |
| rs542482 | rs209139 |  | rs2074478 | rs12190656 | rs2523399 | rs362521 | rs7761965 |
| rs9275601 | rs209152 |  | rs2076169 | rs12210092 | rs2523535 | rs3734523 | rs7765810 |
| rs9277052 | rs209160 |  | rs2233952 | rs12216125 | rs2523864 | rs3762013 | rs7770139 |
| rs9375721 | rs209181 |  | rs2239839 | rs1233374 | rs2524005 | rs3763349 | rs7770216 |
|  | rs2206040 |  | rs2248462 | rs1233384 | rs2524073 | rs3778638 | rs7774407 |
|  | rs2206041 |  | rs2248617 | rs1233386 | rs2524089 | rs3829963 | rs805262 |
|  | rs2233965 |  | rs2267668 | rs1233391 | rs2524123 | rs3873385 | rs86567 |
|  | rs2269553 |  | rs2282851 | rs1233397 | rs2596464 | rs3893464 | rs887464 |
|  | rs2277078 |  | rs2516424 | rs12525269 | rs2596501 | rs3915971 | rs9261387 |
|  | rs2894066 |  | rs2516509 | rs1264344 | rs2621330 | rs3948793 | rs9263726 |
|  | rs3116817 |  | rs2516513 | rs1265109 | rs2735076 | rs396243 | rs9264868 |
|  | rs3116837 |  | rs2523647 | rs13212534 | rs2747430 | rs403414 | rs9264869 |
|  | rs3117143 |  | rs2523710 | rs13437000 | rs2844479 | rs411326 | rs9264904 |
|  | rs3117326 |  | rs2857106 | rs1367731 | rs2844787 | rs423639 | rs9264916 |
|  | rs3117328 |  | rs2905722 | rs1436307 | rs2855430 | rs4386816 | rs9264942 |
|  | rs3129105 |  | rs3093993 | rs1611350 | rs2860580 | rs4713462 | rs9276991 |
|  | rs3129106 |  | rs3095227 | rs169219 | rs2876529 | rs4713607 | rs9276994 |
|  | rs3129791 |  | rs3099844 | rs171329 | rs2894207 | rs4713610 | rs9277912 |
|  | rs3130718 |  | rs3130100 | rs17576984 | rs2905747 | rs4713998 | rs9277932 |
|  | rs3130764 |  | rs3130257 | rs17839997 | rs29232 | rs4713999 | rs9295676 |
|  | rs3130778 |  | rs3130637 | rs1799910 | rs3025642 | rs4714000 | rs9295947 |
|  | rs3130817 |  | rs3132454 | rs1810472 | rs3025643 | rs512808 | rs9295986 |
|  | rs3130826 |  | rs3828893 | rs1865760 | rs3025646 | rs527870 | rs9366778 |
|  | rs3130827 |  | rs456261 | rs1883414 | rs3094054 | rs559818 | rs9368675 |
|  | rs3130830 |  | rs4713600 | rs199736 | rs3094097 | rs576809 | rs9368677 |
|  | rs3130837 |  | rs6940511 | rs199738 | rs3094204 | rs6457699 | rs9368758 |
|  | rs3131093 |  | rs9267139 | rs199741 | rs3094626 | rs6457702 | rs9378200 |
|  | rs3135322 |  | rs9501522 | rs2009610 | rs3095340 | rs6457713 | rs9380326 |
|  | rs422331 |  | rs991760 | rs2013063 | rs3095341 | rs6901221 | rs9380343 |
|  | rs4324798 |  |  | rs2051538 | rs3095352 | rs6905949 | rs9391714 |
|  | rs4713411 |  |  | rs2051541 | rs3117004 | rs6906576 | rs9391858 |
|  | rs4713412 |  |  | rs2070121 | rs3117008 | rs6906846 | rs9468932 |
|  | rs4896678 |  |  | rs2071556 | rs3117016 | rs6910741 | rs9791312 |
|  | rs6904130 |  |  | rs2074483 | rs3117039 | rs6923832 | rs984802 |
|  | rs6918444 |  |  | rs2076483 | rs3128931 | rs6933994 |  |
|  | rs6919321 |  |  | rs2245961 | rs3129214 | rs6940007 |  |
|  | rs6935041 |  |  | rs2246954 | rs3129223 | rs721394 |  |
|  | rs6941262 |  |  | rs2250264 | rs3129234 | rs7382297 |  |
|  | rs7752270 |  |  | rs2256902 | rs3129269 | rs756440 |  |
|  | rs7766902 |  |  | rs2256919 | rs3129274 | rs756441 |  |
|  | rs9257319 |  |  | rs2267633 | rs3130237 | rs7743761 |  |
